# Does Physical Activity Modify the Association between Air Pollution and Recurrence of Cardiovascular Disease?

**DOI:** 10.3390/ijerph18052631

**Published:** 2021-03-05

**Authors:** Wasif Raza, Benno Krachler, Bertil Forsberg, Johan Nilsson Sommar

**Affiliations:** Department of Public Health and Clinical Medicine, Section of Sustainable Health, Umeå University, 901 87 Umeå, Sweden; benno.krachler@umu.se (B.K.); bertil.forsberg@umu.se (B.F.); johan.sommar@umu.se (J.N.S.)

**Keywords:** PM2.5, exercise, active commuting, cardiovascular disease prevention, interaction

## Abstract

We aimed to assess a possible interaction effect between physical activity and particulate air pollution exposure on recurrence of ischemic heart disease (IHD) and stroke. We followed 2221 adult participants comprising first time IHD (1403) and stroke (818) cases from the Västerbotten Intervention Program between 1 January 1990 to 31 December 2013. During mean follow-up times of 5.5 years, 428 and 156 participants developed IHD and stroke recurrence, respectively. PM2.5 concentrations above the median (5.48 µg/m^3^) were associated with increased risk of IHD and stroke recurrence by 13% (95% CI −17–45%) and 21% (95% CI −19–80%), respectively. These risk increases were however only observed among those that exercised at most once a week at 21% (95% CI −5–50%) and 25% (95% CI −19–90%) for IHD and stroke recurrence, respectively. Higher frequency of exercise at recruitment was positively associated with IHD and stroke recurrence but only the association with IHD recurrence among participants with low residential PM2.5 was statistically significant (96% increased risk (95%-CI 22–215%)). However, no interaction effect between physical activity and PM2.5 exposure was found. Our findings suggest that physical activity may reduce the air pollution exposure associated risk for recurrent cardiovascular disease, likely by reducing the inflammatory response.

## 1. Introduction

Air pollution is an important environmental health determinant contributing to disease burden. According to the Global Burden of Disease Study, outdoor air pollution was ranked fifth among modifiable risk factors, above other common risk factors such as physical inactivity and high cholesterol, and contributes to more than 4 million premature deaths yearly worldwide [1]. The adverse health effects of exposure to particulate matter (PM), including particles with a median aerodynamic diameter <2.5 μm (PM2.5) and <10 μm (PM10), are of great concern to governments and health organizations worldwide [2]. Although the relative effects of air pollutants are larger for respiratory events than for cardiovascular disease, the numbers of adverse health outcomes attributable to air pollutants are much larger for cardiovascular disease [3].

Cardiovascular disease is a substantial public health threat and remains the most common cause of death in Europe, accounting for 45% of all deaths [4]. Ischemic heart disease (IHD) and stroke constitute the major diagnoses contributing to the cardiovascular disease burden in terms of both increased morbidity and mortality. Inflammation and oxidative stress are suggested as principal underlying mechanisms for the detrimental effects of air pollution on the cardiovascular system [5]. Patients who have survived a myocardial infarction and stroke are at increased risk for recurrent ischemic events which highlights the importance of developing both effective primary and secondary prevention strategies [6].

The compounded effects of industrial and automotive innovation, as well as other social-cultural changes around the world, have led to increased physical inactivity. Physical inactivity is a major risk factor for mortality and is one of the most important contributors to the global burden of disease [7,8]. Data from 122 countries showed that 31.1% of adults (aged 15 years or older) were physically inactive [9]. The importance of regular physical activity is now well-established, as are the harmful consequences of sedentary lifestyles. There is extensive evidence that physical activity reduces the risk of chronic diseases, including ischemic heart diseases and stroke, through its anti-inflammatory effect [10,11]. It is thus plausible that regular physical activity may counteract the harmful effects of air pollution on cardiovascular disease (CVD) through its anti-inflammatory effects.

Increasing physical activity in the general population is a public health priority. Promotion of walking and cycling for transport (active travel) is a promising strategy to that end, since risks can be reduced for many diseases [12]. However, the increased ventilation rates of pedestrians and cyclists can result in larger doses of inhaled pollutants compared to passive modes of travel [13].

Data from epidemiological studies investigating the long-term impact of air pollution and physical activity on recurrence of CVD are scarce. Findings from the Danish Diet, Cancer and Health cohort study of 1233 participants showed that participation in gardening, cycling and walking was inversely associated with recurrent myocardial infarction (MI) whereas participation in sports increased the risk of recurrent MI [14]. However, all estimates were non-significant and there was no effect modification of association between physical activity and MI by nitrogen dioxide (NO_2_), a marker of vehicle exhaust emissions. To the best of our knowledge, no previous study has investigated the combined impact of air pollution and physical activity on recurrent stroke.

This study’s objective is to estimate the risk of recurrent CVD associated with residential air pollution exposure and physical inactivity, and assess possible interaction effects.

## 2. Materials and Methods

### 2.1. Cohort Description

Our study period ranged from 1 January 1990 to 31 December 2013 and included individuals from Västerbotten Intervention Program (VIP) living in Umeå municipality, Sweden. VIP is a population-based health screening and intervention program to reduce premature cardiovascular disease and was initiated in year the 1985. All inhabitants living in the County of Västerbotten were invited to their local primary health care center for a health examination and counseling in the years they turned 40, 50 and 60 years of age. The participants completed an extensive questionnaire analyzing physical activity, education, smoking, occupation and alcohol intake. Written informed consent was obtained from all VIP participants before enrolment in the study. This study, with the addition of air pollution data, was approved by the Regional Ethical Committee at Umea University (DNR: 2014-136-32M and 2015/16-31Ö). A detailed description of VIP may be found elsewhere [15]. All cohort members in Umeå municipality who, according to the regional patient register, survived a first incidence IHD or stroke during the study period were eligible for this study.

### 2.2. Physical Activity

The VIP questionnaire included questions on both leisure time physical activity and active commuting (travelling to and from work). The participants were asked about the frequency of exercise in training clothes during the previous three months and activity level was categorized as low (if they exercised never, rarely or once per week) or high (if they exercised 2–3 times per week or more than 3 times per week). Information on active commuting was collected by inquiring about the mode of travelling to work in each of the four seasons with answering options of travelling by car, bus, walking and cycling. Participants were classified as less frequent active commuters if they commuted by car or bus, or by walking and cycling at most two seasons out of four, or as frequent active commuters if they walked or cycled to work more than two seasons out of four.

### 2.3. Air Pollution Assessment

The details of air pollution assessments have been described elsewhere [16]. In brief, PM2.5 estimates were obtained from the Swedish Clean Air and Climate Research Program (SCAC). Air pollution estimates were calculated in three stages for Umeå. In a first stage, locally generated particle concentrations as PM10 and PM2.5 were estimated by applying high resolution dispersion modeling on source-specific local emission inventories for the years 1990, 2000 and 2011 and thereafter extrapolated linearly up to 2014. Emission factors for traffic exhaust were taken from inventory data on different vehicle types, speeds and driving conditions based on the Handbook on Emission Factors for Road Traffic version 3.1 [17]. Non-exhaust contributions from brake and tire wear were calculated by using methods previously described elsewhere [18,19]. Information on small-scale residential heating was taken from data of chimney sweepers and interviews about amount of wood burning [16] whereas data from the Swedish national emission inventory [20] and from environmental permits and emission reports were used to estimate emissions from industrial sources and the national Swedish inventory was used to estimate emissions from off-road machinery and agricultural sources. Modeled estimates were validated with measured estimates at monitoring stations with values of r^2^ = 0.87 and r^2^ = 0.65 for PM10 and PM2.5, respectively [16].

In a second stage, long-term transported PM10 and PM2.5 concentrations were estimated by calculating the difference between measured concentrations at monitoring stations and modeled local particle concentrations at the same location, taking into account hourly meteorological data. Thereafter, annual averages of long-range contributions were added to the dispersion model estimates of local emissions to obtain total PM10 and total PM2.5 concentrations. Finally, modeled average concentrations of total PM10 and PM2.5 were assigned to each participant.

For this study, we used annual mean concentrations of PM2.5 one year prior to the recurrence of IHD and stroke to estimate participants’ long-term PM2.5 exposure. For the analysis, participants were divided into those exposed to high (above the 50th percentile of exposure range) or low (below the 50th percentile of exposure range) residential PM2.5 concentrations.

### 2.4. Health Outcomes

IHD or stroke after more than 28 days from the recorded first incident IHD or stroke episode was considered as a recurrent IHD or stroke event, respectively. The International Classification of Diseases Ninth Revision (ICD-9) codes 410–414 and ICD-10 I20-25 were used to define IHD and ICD-9 codes 431–436 and ICD-10 codes I61–I65 to define stroke [21,22]. Data on hospitalization and mortality due to IHD and stroke were retrieved through linkage of participants to well-validated in-patient and cause of death registers of the Swedish National Board of Health and Welfare [23] by their unique Swedish personal identification number.

### 2.5. Statistical Methods

Cox proportional hazard models were used to investigate the effect of air pollution and physical activity on the recurrence of IHD and stroke in our study population. Air pollution was assessed as the annual mean residential PM2.5 concentration one year preceding the event. Age, being the most important confounder, was used as the underlying time scale. All individual covariates were collected at recruitment whereas air pollution exposure was modeled yearly. Study subjects’ deaths from causes other than IHD and stroke were censored, as were those who permanently emigrated from the study area before the end of the study period. Associations were adjusted for gender, calendar year, smoking status (never smoker, previous non-regular smoker, non-regular smoker, cigarette smoker, cigar or pipe smoker), alcohol intake (never, once/month or sometimes, 2–4 times/month, 2–3 times/week, ≥4 times/week), highest education level (compulsory, high school, university), occupation status (gainfully employed, unemployed/not gainfully employed, retired) and registry data on area level mean income.

Associations were assessed separately for recurrent IHD and stroke.

Firstly, overall associations with air pollution and physical activity (frequency of exercise/active commuting) were estimated without incorporating any interaction terms. Secondly, a multiplicative interaction was incorporated to estimate associations with exercise/active commuting among individuals with high/low (above/below median) residential air pollution as well as associations with air pollution among less and more frequently physically active individuals.

To evaluate whether time duration between recruitment and first incidence of disease affects the association between physical activity and disease recurrence we also assessed categories of pre-incident follow-up (<8, 8–16 and >16 years). In the subset of the IHD and stroke patients that had undergone two cohort examinations, the impact of change in physical activity was also assessed.

Within VIP, 1680 VIP cohort members with first incident IHD and 985 with a first incident stroke were eligible for the study. Among these, we excluded participants if they had missing information on PM2.5 (79), exercise (69) and active commuting (361), leaving 2221 participants with data for analysis. Of these, 1403 and 818 were first incident IHD and stroke cases, respectively.

All analyses were performed using R version 3.4.2 [24] and the statistical inference was conducted with a 5% significance level.

## 3. Results

Mean ages of first incident stroke and IHD were 52 and 53 years, respectively. A total of 325 (23%) and 385 (47%) of incident cases were women, respectively. During the period of follow-up, starting at first incidence, 428 of the 1403 subjects in the IHD subgroup had a recurrence of IHD and 156 of the 818 in the stroke subgroup had a recurrence of stroke. Average follow-up time was 5.5 years, respectively. For the IHD subgroup, PM2.5 concentrations were on average higher among the individuals with low activity level, compared with those exercising twice per week or more (Table 1). The average time to recurrence of IHD and stroke was shorter among those who exercised twice per week or more. Individuals in the IHD group who, at baseline examination, exercised twice per week or more also had a higher probability of frequent active commuting and higher level of education, and were less likely to be smokers. No differences were observed, however, regarding gainful employment and alcohol consumption. Similar tendencies were observed in the stroke subgroup. Median concentration of residential PM2.5 was 5.48 µg/m^3^ in participants who developed recurrent MI and stroke during follow-up. No significant difference in average commuting distance was observed due to difference in residential particle exposure (Table S1).

### 3.1. Association with Air Pollution at Different Levels of Exercise

**Figure 1:**
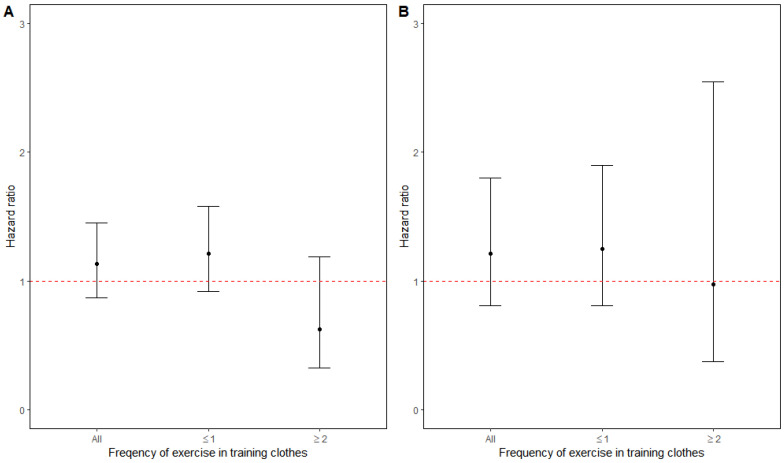
Adjusted hazard ratios (95% CI) for (**A**) IHD and (**B**) stroke recurrence associated with high compared with low air pollution, overall and in categories of exercise in training clothes.

We observed positive but non-significant associations between long-term PM2.5 exposure and recurrence of IHD and stroke. After adjusting for potential confounders, high residential PM2.5 concentrations were associated with 13% and 21% increased risks of IHD and stroke, respectively (Table 2 and Figure 1). These increased risks were however only observed among the individuals with low levels of physical activity at baseline (21% and 25%, respectively). Among the individuals with a higher frequency of exercise, we observed risk reductions for recurrent IHD and stroke by 38% and 3%, respectively. Similar results were obtained for recurrence of IHD when comparing PM2.5 risk estimates between frequent and less frequently active commuters. Higher PM2.5 concentrations were, however, associated with increased risk of recurrent stroke both among frequent and less frequently active commuters (10% and 46% increased risk, respectively). None of these risk ratios were statistically significant.

### 3.2. Association with Physical Activity at Different Levels of PM2.5

**Figure 2:**
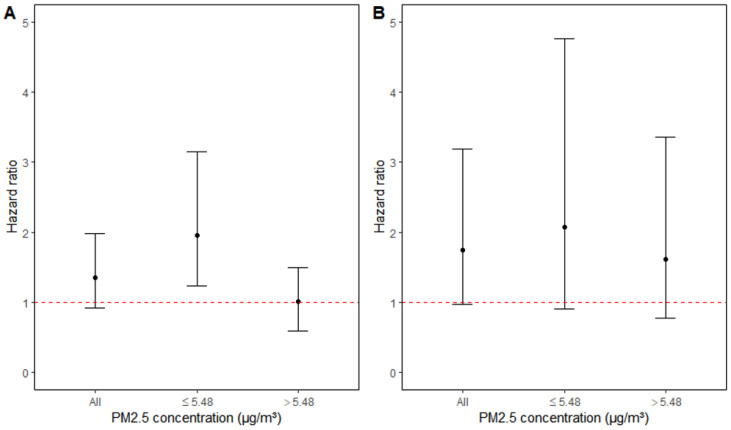
Adjusted hazard ratios (95% CI) for (**A**) IHD and (**B**) stroke recurrence associated with exercise in training clothes, overall and in categories of PM2.5 concentrations.

Exercise at least twice a week at baseline was associated with increased risk estimates for both recurrent IHD and stroke. The risk increased by 35% and 75%, respectively (Table 3 and Figure 2). The risk increase for recurrent IHD was, however, only observed among those with low PM2.5 exposure. A statistically significant 96% increased risk was found among those who at baseline exercised at least twice a week. The risk increase for recurrent stroke was similar, but was found in both exercise groups. Neither, however, were statistically significant. Associations with active commuting were less apparent.

An additional analysis among 514 IHD patients and 253 stroke patients with two cohort examinations showed that the observed high risks of disease recurrence associated with exercise were mainly driven by risk increases among those participants who had decreased the volume of habitual exercise (Table S2). The time duration between recruitment and first incidence of disease did not modify the risk increase associated with frequency of exercise at baseline (Table S3).

## 4. Discussion

We observed increased risks of recurrent IHD and stroke associated with higher levels of air pollution exposures at home addresses, but no protective effect of physical activity. Air pollution-associated risks were, however, only observed among those who exercised at most once a week. Higher frequency of exercise at recruitment was found to be associated with an increased risk of recurrent IHD and stroke. Increased risk of recurrent IHD was, however, only observed among individuals with low residential PM2.5 exposure. This increased risk also reached statistical significance, but was not statistically significant different from the change in risk among the highly exposed. Associations with active commuting at baseline were less apparent.

Systemic inflammation along with hemostatic markers have been suggested as the main mediating mechanisms for the beneficial effect of physical activity on the risk for cardiovascular disease [25]. We hypothesized that the anti-inflammatory effect of physical activity may counteract the harmful effect of air pollution on recurrence of IHD and stroke. Our observed decreased air-pollution-associated change in risk for IHD and stroke recurrence among more physically active participants supports this hypothesis. This modification of the air-pollution-associated effect was also observed by active commuting for IHD recurrence, but not for recurrent stroke. A modifying effect in the opposite direction was, however, observed in a Danish cohort [14]. However, none of these interaction effects reached statistical significance. Although our study was fairly large, there were a limited number of participants who exercised in training clothes more than once a week.

The fact that participants with high levels of physical activity at baseline had an increased risk for recurrent IHD and stroke may appear counterintuitive. Nevertheless, the data show that exposure to protective levels of a modifiable risk factor during a first-time event is expected to become a liability when recurrence is studied: individuals with high level of activity have less scope to reduce their risk than their inactive peers. Moreover, as the average time to disease recurrence was short, 142 days for IHD and 157 days for stroke, those who had been active prior to first incidence may not have resumed exercising and thus increased their risk for disease recurrence. In sub-analyses of individuals with two health examinations (10 years apart), the increased risk of stroke recurrence associated with higher level of physical activity was found only among those that had reduced their physical activity (from high to low, as previously defined). Such a tendency was also observed for IHD recurrence. The subsample was however too small to be able to show any statistically significant differences.

No apparent association was found between active commuting and disease recurrence. An 11% increased risk was observed for recurrent stroke, which was driven by a 23% increased risk among individuals with higher air pollution exposure. Cycling and walking distances in men and women were longer among less frequently active commuters but no statistically significant difference in distance was found between low and highly exposed individuals; however, there was a tendency towards longer distances among the highly exposed (Table S1).

As far as we know, only one previous cohort study has assessed the combined long-term effects of physical activity and air pollution on recurrence of cardiovascular disease [14]. Consistent with our findings, the Danish Diet, Cancer and Health cohort study, comprising 1233 individuals with incident MI, reported an increased but not statistically significant risk of recurrent MI associated with participation in sports. Participation in sporting activities for between half an hour to four hours per week and more than four hours per week increased the risk of MI recurrence by 3% and 15% respectively. The increased risk in their study was, however, only present among individuals within the second and third tertile of NO2 exposure at the home address (25% increased risk among those with medium (14.3 to 21.0 µg/m^3^) and high (≥21.0 µg/m^3^) exposure). In contrast to our results, they observed a reduced risk of recurrent MI among the least exposed (<14.0 µg/m^3^ NO_2_).

Our results cannot be directly compared with the Danish cohort due to a difference in pollutants and air pollution concentration levels [26]. Annual mean concentration of PM2.5 reported in a recent study within the same Danish cohort was about twice as high as our estimate, although interquartile ranges (IQRs), which were more driven by local sources, were almost similar between the cohorts. Despite this comparatively lower total level of air pollution, an increased risk of recurrent IHD and stroke was observed in our study but not among those who exercised at least twice a week.

Contrary to the findings in this and the Danish study, Hållmarker et al. (2015) reported a 32% (95% CI 13–47%) lower risk of recurrent MI when comparing previous participants in a long-distance ski race with non-participants; age-specific mortality was reduced by 29% (95% CI 12–43) but no risk reduction was found for recurrent stroke [27,28]. Compared with the highly physically active individuals in our study, these long-distance skiers may have been more physically active and may also have had a healthier lifestyle in general; from questionnaires, 60% of the skiers exercised at least 4 h per week [29].

The strength of this study lies in the prospective study design, the availability of a number of important confounding factors at the year of inclusion into the cohort, and the detailed data on air pollution concentrations. Compared to a previous study, our study uses high resolution dispersion modeled particle concentrations by annual mean concentrations during the period of follow-up using the population address registry to account for changes in addresses. The outcome was recorded using comprehensive national patient and cause of death registries. The analyses also included information about changes in physical activity for a subset of the individuals and sensitivity analyses in categories of follow-up time were also conducted.

Our study also has several limitations that need to be acknowledged. Firstly, due to statistical power the study was not able to use more than two exposure categories and was therefore not able to demonstrate any dose–response relationships. The a priori univariate power analysis showed that 985 cases of disease recurrence would be needed to show a statistically significant hazard ratio of 1.25 with 20% of the individuals in the highly physical activity group, requiring 80% statistical power with a 5% significance level for a two-sided statistical test. For air pollution particle concentrations dichotomized by the median, 631 cases would be required for the same effect size. We did however only observe 10% of the incident IHD and stroke cases in the high physical activity group. Secondly, the study considered only frequency of exercise and active commuting since detailed information about intensity and duration was not available from the questionnaire. Furthermore, differences in physical activity intensity may be hypothesized comparing groups with high and low residential PM2.5 concentration, due to different types of activities. The study also lacked information on whether exercise mainly took place outdoors or indoors. Thirdly, there is risk of reverse causation if individuals had low physical activity at baseline due to poor health with initial increased risk of cardiovascular disease. Similarly, disease occurrence during follow-up may also reduce the initially reported physical activity. Fourthly, although the estimates were adjusted for several potential confounders that may affect the associations, some residual confounding by, for instance, the air pollution exposure during commuting, can still be present.

## 5. Conclusions

This study indicates a higher risk of recurrent IHD and stroke among individuals with higher residential particle concentrations, but this increased risk is only present among individuals that exercise less frequently. Exercise at recruitment was also associated with increased risks of recurrent IHD and stroke, where the higher risk was mainly observed among individuals with low particle concentration at their home address. These modifications of associations are in the direction of the hypothesis that physical activity may reduce the inflammatory response to air pollution exposure and thereby reduce the risk of attributable disease. These interaction effects were, however, not statistically significant and further studies are needed to confirm the findings.

## Figures and Tables

**Table 1:** Characteristics of participants at different levels of exercise in training clothes at baseline.

Variables	At Most Once a Week	Twice per Week or More	*p*-Value	At Most Once a Week	Twice per Week or More	*p*-Value
	IHD			Stroke		
Number of incident cases	1261	142		731	87	
Number of recurrent cases	382	46		136	20	
Time to disease recurrence (days) (mean (SD))	161.04 (107.20)	142.32 (107.67)	0.05	168.43 (107.80)	156.92 (112.17)	0.35
PM2.5 (mean (SD))	5.57 (0.74)	5.60 (0.80)	0.39	5.80 (0.89)	5.62 (0.83)	0.02
Age, years (mean (SD))	52.11 (7.80)	51.20 (8.29)	0.19	53.31 (7.74)	51.72 (9.18)	0.08
Frequency of active commuting (%)			0.05			0.96
At most two seasons of four	920 (73.0)	92 (64.8)		489 (66.9)	59 (67.8)	
More than two seasons of four	341 (27.0)	50 (35.2)		242 (33.1)	28 (32.2)	
Gender (% women)	347 (27.5)	31 (21.8)	0.18	293 (39.9)	32 (36.8)	0.65
Alcohol intake (%)			0.60	291 (39.8)	32 (36.8)	0.94
Never	5 (0.4)	1 (0.7)		2 (0.3)	0 (0.0)	
Once/month or sometimes	373 (29.6)	41 (28.9)		223 (30.5)	25 (28.7)	
2–4 times/month	222 (17.6)	17 (12.0)		102 (14.0)	12 (13.8)	
2–3 times/week	6 (0.5)	1 (0.7)		4 (0.5)	1 (1.1)	
≥4 times/week	14 (1.1)	2 (1.4)		0	0	
Missing	641 (50.8)	80 (56.3)		400 (54.7)	49 (56.3)	
Smoking (%)			0.03			0.11
Never smoker	451 (35.8)	54 (38.0)		303 (41.5)	41 (47.1)	
Previous non-regular smoker	93 (7.4)	14 (9.9)		53 (7.3)	8 (9.2)	
Non-regular smoker	55 (4.4)	11 (7.7)		27 (3.7)	3 (3.4)	
Previous regular smoker	306 (24.3)	39 (27.5)		185 (25.3)	25 (28.7)	
Cigarette smoker	299 (23.7)	16 (11.3)		145 (19.8)	6 (6.9)	
Cigar or pipe smoker	26 (2.1)	3 (2.1)		12 (1.6)	2 (2.3)	
Missing	31 (2.5)	5 (3.5)		6 (0.8)	2 (2.3)	
Highest education level (%)			0.06			0.07
Compulsory	715 (56.7)	70 (49.3)		438 (59.9)	40 (46.0)	
High	216 (17.1)	21 (14.8)		119 (16.3)	21 (24.1)	
University	305 (24.2)	49 (34.5)		1721(23.4)	26 (29.9)	
Missing	25 (2.0)	2 (1.4)		3 (0.4)	0 (0.0)	
Occupation (%)			0.92			0.23
Gainfully employed	1075 (85.2)	124 (87.3)		662 (90.6)	73 (83.9)	
Unemployed	37 (2.9)	3 (2.1)		9 (1.2)	1 (1.1)	
Not gainfully employed	14 (1.1)	1 (0.7)		4 (0.5)	1 (1.1)	
Retired	38 (3.0)	3 (2.1)		20 (2.7)	6 (6.9)	
Missing	97 (7.7)	11 (7.7)		36 (4.9)	6 (6.9)	
Mean income for the neighborhood (SEK) (mean (SD))	130,609 (21,727)	133,386 (20,993)	0.15	129,489 (21,238)	132,431 (20,219)	0.22

**Table 2:** Adjusted hazard ratios (95% CI) for IHD (ischemic heart disease) and stroke recurrence associated with high air pollution levels vs. low at the home address among persons with different exercise/commuting habits.

Outcome/Exposure	Overall Model Hazard Ratios with no Interaction Effects	^a^ Hazard Ratios in Different Exercise Categories		^a^ Interaction Hazard Ratios
		Exercise in training clothes		
IHD		≤once/week	≥twice/week	
Low PM_2.5_^b^	1	1	1	
High PM_2.5_^b^	1.13 (0.87–1.45)	1.21 (0.92–1.58)	0.62 (0.32–1.19)	0.51 (0.26–1.02)
Stroke				
Low PM_2.5_^b^	1	1	1	
High PM_2.5_^b^	1.21 (0.81–1.80)	1.25 (0.81–1.9)	0.97 (0.37–2.55)	0.78 (0.28–2.19)
		Active commuting		
IHD		≤two seasons of four	>two seasons of four	
Low PM_2.5_^b^	1	1	1	
High PM_2.5_^b^	1.13 (0.87–1.45)	1.23 (0.92–1.64)	0.89 (0.58–1.35)	0.72 (0.45–1.16)
Stroke				
Low PM_2.5_^b^			1	
High PM_2.5_^b^	1.21 (0.81–1.80)	1.1 (0.68–1.76)	1.46 (0.77–2.74)	1.33 (0.64–2.77)

^a^ Adjusted for sex, calendar year year, education, smoking, alcohol intake, occupation, neighborhood mean income, leisure time physical activity and active commuting. ^b^ Low PM2.5: ≤5.48 µg/m^3^; high PM2.5: >5.48 µg/m^3^.

**Table 3:** Adjusted hazard ratios (95% CI) for IHD and stroke recurrence associated with different exercise and commuting habits among persons with different air pollution exposures at home addresses.

Exercise in Training Clothes	Overall Model Hazard Ratios with no Interaction Effects	^a^ Hazard Ratios in Categories of High and Low Particle Exposure	
IHD		Low PM2.5 ^b^	High PM2.5 ^b^
≤once/week	1	1	1
≥twice/week	1.35 (0.92–1.98)	1.96 (1.23–3.15)	1.01 (0.59–1.72)
Stroke			
≤once/week	1	1	1
≥twice/week	1.75 (0.97–3.19)	2.07 (0.90–4.76)	1.61 (0.77–3.36)
Active commuting per season		Low PM2.5 ^b^	High PM2.5 ^b^
IHD			
≤two seasons of four	1	1	1
>two seasons of four	0.96 (0.75–1.25)	1.18 (0.82–1.71)	0.86 (0.62–1.19)
Stroke			
≤two seasons of four	1	1	1
>two seasons of four	1.11 (0.75–1.65)	0.93 (0.51–1.70)	1.23 (0.76–2.01)

^a^ Adjusted for sex, calendar year, education, smoking, alcohol intake, occupation, neighborhood mean income, leisure time physical activity and active commuting. ^b^ Low PM2.5: ≤5.48 µg/m^3^; high PM2.5: >5.48 µg/m^3^.

## Data Availability

No additional data are available.

## Supplementary Materials

The following are available online at https://www.mdpi.com/1660-4601/18/5/2631/s1. Table S1. Average distance of active commuting trip (single) from home to working place among participants with low and high particle exposure, Table S2. Association between assessment in exercise between two health examinations and IHD/stroke recurrence, Table S3. Association between exercise and IHD/stroke recurrence among participants with different time from screening giving the eligibility to first incident IHD/stroke.
