# Supplementary material for: Does Physical Activity Modify the Association between Air Pollution and Recurrence of Cardiovascular Disease?
Source: Int J Environ Res Public Health. 2021 Mar 5;18(5):2631. doi: 10.3390/ijerph18052631 (PMC7967368; doi:10.3390/ijerph18052631)
Supplement: Supplementary file 1 [file ijerph-18-02631-s001.pdf]

Supplementary material

Table S1. Average distance of active commuting trip (single) from home to working place among participants with low and high particle exposure.

IHD	Male			Female		
**Active commuting (km per trip) (n, mean (SD))**	Low PM2.5	High PM2.5	p-value	Low PM2.5	High PM2.5	p-value
**One season**	124, 7.14 (9.47)	193, 5.64 (7.54)	0.14	77, 4.25 (5.31)	107, 5.59 (8.73)	0.20
**At most two seasons of four**	46, 9.70 (11.2)	76, 7.56 (9.37)	0.28	31, 6.39 (9.72)	40, 8.22 (11.2)	0.41
**More than two seasons of four**	87, 5.36 (7.60)	108, 4.50 (6.02)	0.40	47, 2.76 (2.74)	66, 4.08 (4.35)	0.13
**Stroke**						
**Active commuting (km per trip) (n, mean (SD))**						
**One season**	64, 4.75 (4.48)	108, 6.34 (9.98)	0.15	77, 5.18 (7.11)	96, 5.13 (8.98)	0.96
**At most two seasons of four**	28, 6.46 (5.78)	40, 10.1 (13.3)	0.13	37, 6.62 (9.58)	32, 8.84(13.1)	0.43
**More than two seasons of four**	36, 3.42 (2.49)	68, 4.12 (6.53)	0.43	40, 3.85 (3.15)	64, 3.27 (5.16)	0.48

Table S2. Association between assessment in exercise between two health examinations and IHD/stroke recurrence

Exercise in training clothes	[a] HR (95% CI)	Number of participants
**IHD**		
**Low-Low**	1	367
**High-High**	1.26 (0.51-3.09	25
**Low-High**	1.12 (0.67-1.89)	95
**High-Low**	1.62 (0.65-3.99)	27
**Stroke**		
**Low-Low**	1	179
**High-High**	0 (0-0)	19
**Low-High**	0.91 (0.32-3.6)	47
**High-Low**	2.68 (0.52-28.44)	8

[a]Adjusted for sex, calendar year, education, smoking, alcohol intake, occupation, leisure time physical activity, active commuting, and neighbourhood mean income

Table S3. Association between exercise and IHD/stroke recurrence among participants with different time from screening giving the eligibility to first incident IHD/stroke

**Exercise in training clothes**	**Adjusted[a] HRs in different categories of time duration**		
**IHD**	Short	Medium	Long
**≤ once/week**	1	1	1
**≥ twice/week**	1.14 (0.67-1.94)	1.85 (1.03-3.32)	1.11 (0.46-2.69)
**Stroke**			
**≤ once/week**	1	1	1
**≥ twice/week**	1.66 (0.59-4.69)	1.57 (0.73-3.38)	2.69 (0.48-15.17)

[a]Adjusted for sex, calendar year, education, smoking, alcohol intake, occupation, leisure time physical activity, active commuting, and neighbourhood mean income
